# Diagnostic Potential of Salivary Interleukin-1*β* and IL-10 for Distinguishing Periodontal Health From Periodontitis and Stable From Unstable Periodontitis: A Case–Control Study

**DOI:** 10.1155/2024/8006278

**Published:** 2024-10-15

**Authors:** Zainab J. Raheem, Hayder Raad Abdulbaqi

**Affiliations:** Department of Periodontics, College of Dentistry, University of Baghdad, Baghdad, Iraq

**Keywords:** biomarkers, IL-1*β*, IL-10, periodontal health, periodontitis

## Abstract

**Objective:** This case–control study aimed to investigate the diagnostic accuracy of salivary interleukin (IL)-1*β*, IL-10, and IL-1*β*/IL-10 ratio to discriminate periodontitis from periodontal health and stable from unstable periodontitis.

**Methods:** Saliva samples were collected from 135 (healthy on an intact periodontium = 45 [as healthy control], stable periodontitis = 39, and unstable periodontitis = 51) participants, and then clinical periodontal parameters were recorded. An enzyme-linked immunosorbent assay was used to determine salivary levels of IL-1*β* and IL-10. Area under the curves (AUCs), sensitivity, and specificity of IL-1*β*, IL-10, and IL-1*β*/IL-10 were estimated to discriminate between groups.

**Result:** The level of salivary IL-1*β* was significantly higher in unstable periodontitis than in stable periodontitis and healthy control groups (426 ± 59, 247 ± 55, and 204 ± 36 pg/ml [picograms per milliliter], respectively). While the level of salivary IL-10 was significantly higher in the control group (360.7 ± 80.5 pg/ml) than unstable periodontitis group (146.92 ± 1.8 pg/ml), no significant difference was found between the control and stable periodontitis (317.04 ± 59.8 pg/ml) groups. IL-1*β*, IL-10, and IL-1*β*/IL-10 had significant diagnostic accuracy for differentiating healthy control from unstable periodontitis (AUCs = 0.99, 0.96, and 1; sensitivity = 0.98,1, and 1; specificity = 0.95, 0.95, and 1, respectively). Similarly, they showed significant diagnostic accuracy in distinguishing unstable from stable periodontitis (AUCs = 0.98, 0.99, and 1; sensitivity = 0.94, 1, and 1; specificity = 0.94, 0.97, and 1, respectively).

**Conclusion:** Salivary IL-1*β*, IL-10, and IL-1*β*/IL-10 have a high potential to discriminate healthy control from periodontitis and stable from unstable periodontitis.

**Trial Registration:** ClinicalTrials.gov identifier: NCT05722613

## 1. Introduction

Periodontitis is a common chronic multifactorial inflammatory disease [1]. Globally, 796 million individuals are suffering from periodontitis; the prevalence of severe periodontitis is 9.8%. With increases in age, these numbers are expected to rise [2]. Periodontitis has a negative impact on the quality of life associated with oral health [3]. Moreover, periodontitis has been reported to be associated with several systemic disorders, such as dementia [4], diabetes, and cardiovascular diseases [5]. Accordingly, early and precise diagnosis of periodontitis is crucial since the loss of soft tissue and bone could have esthetic, functional, and systemic consequences [6].

The main cause of periodontitis is the shifting in the ecological balance between virulent periodontal pathogens and the host immune response [7]. The inflammatory changes in periodontal tissue are initiated by dental biofilm (harboring many periodontal pathogens) accumulated on teeth surfaces [8]. Host inflammatory cells response to virulent pathogens and release both pro-inflammatory and anti-inflammatory cytokines [9]. Interleukin (IL)-1*β* is a pro-inflammatory cytokine associated with tissue degradation in periodontitis [10]. Elevation in its levels could be detected shortly in the gingival crevicular fluid after the initiation of gingival inflammation [11]. It is released from macrophages, monocytes, lymphocytes, epithelial cells, fibroblasts, and osteoblasts in response to periodontal pathogens. IL-1*β* has been reported to be elevated in serum, saliva, and gingival crevicular fluid of periodontitis patients [12, 13]. After nonsurgical periodontal therapy, salivary levels of IL-1*β* are significantly reduced [14]. On the other hand, regulatory T cells release IL-10 in the affected tissue. IL-10, known as an anti-inflammatory cytokine, plays a complex role in the pathogenesis of periodontitis. IL-10 declines alveolar bone loss by downregulation of osteoclastogenesis mediated by T-helper 1 [15]. Simultaneously, IL-10 suppresses the innate response [16]. Thus, the role of IL-10 is to control the immune response and hinder the production of pro-inflammatory cytokines. For instance, IL-10 has the ability to restrict and inhibit the action of IL-1*β* [17]. Thus, it has a protective role in reducing inflammatory-driven alveolar bone resorption and tissue damage [18]. Consequently, the overall pro- and anti-inflammatory balance could be able to identify whether the lesions are stable or progressing [19].

In fact, accurate diagnosis of periodontal diseases is essential for appropriate treatment planning as well as prognosis [20]. No doubt, the precision of the diagnosis of periodontal diseases not only impacts clinical practice but also boosts the validity and reliability of research aimed to enhance periodontal care strategies [21].

Currently, clinical parameters such as bleeding on probing (BOP), clinical attachment loss (CAL), probing pocket depth (PPD), and radiographs are still the standard approaches for diagnosing periodontal diseases. By these parameters, clinicians could assess the present extent and severity of periodontitis as well as illustrate the past tissue destruction of periodontal tissue [22]. However, the above-mentioned parameters have several considerable limitations. They are deficient in providing data about the current biological activity or future progression of the disease [22]. Additionally, the accuracy of measuring these parameters is more dependent on clinician skills. Therefore, there are marginal errors in recording these parameters among different clinicians [23]. This might have an impact on the precision of diagnosing periodontal diseases. For instance, the current status of periodontitis, according to the recent classification, is detected after recording BOP and PPD [20]. In light of these limitations, the recent classification of periodontal diseases has brought attention to the possible use of biomarkers to enhance the early and accurate diagnosis of periodontitis [20].

The nature of periodontitis is intricate, and it is inadequate to rely on a solitary biomarker to encompass all dimensions of the disease [24]. Identifying a prospective panel consisting of combined salivary biomarkers exhibits an enhanced efficacy in differentiating patients with varying stages and statuses of periodontitis from healthy peers. Therefore, this study aimed to determine the diagnostic accuracy of salivary levels of tissue destruction-associated biomarkers (IL-1*β* and IL-10) for differentiating periodontal health from periodontitis and stable from unstable periodontitis.

## 2. Methods

### 2.1. Study Design and Population

This observational case–control study was conducted at the Department of Periodontics/College of Dentistry/University of Baghdad and Al-Noor Specialized Dental Center from May 2022 to March 2023. This study followed the ethical guidelines of the Declaration of Helsinki and its revisions for human research. The protocol has been approved by the ethics committee of the College of Dentistry/University of Baghdad (Reference number: 527, Project number: 527622 on April 17th, 2022).

During screening, only subjects who fit the inclusion and exclusion criteria were invited to participate in this study. Signed informed consent forms were collected by the examiner from all participants.

The inclusion criteria included those who were systemically healthy with body mass index (BMI) ≤25, not under any medications in the last 3 months and had a minimum of 20 teeth with facial and lingual scorable surfaces. Smoker subjects or subjects having diabetes were excluded from this study. Additionally, exclusion criteria included any previous periodontal therapy within the last 3 months or currently under active periodontal treatment. Accordingly, the participants were diagnosed and categorized into three groups: healthy on an intact periodontium as a healthy control, unstable periodontitis, and stable periodontitis. In the control group, the participants were diagnosed as having healthy periodontium characterized by BOP <10%, PPD ≤3 mm, and intact periodontium with no probing attachment loss [25]. On the other hand, participants having periodontitis were diagnosed as they were having interdental CAL detected at ≥2 nonadjacent teeth or/and facial or oral surfaces with CAL ≥3 mm as well as PPD >3 mm detected at ≥2 teeth [26]. In unstable periodontitis group, the participants were diagnosed as generalized (>30% of teeth having CAL) unstable periodontitis having at least one site with PPD ≥5 mm or PPD ≥4 mm with BOP. While stable periodontitis group included participants having generalized stable periodontitis with BOP <10%, PPD ≤4 mm, and no BOP at 4 mm PPD sites [26]. Both stable and unstable periodontitis groups had a history of active periodontal treatment and were currently in the maintenance phase.

A pilot study was conducted to optimize the techniques and compute the required sample size. After estimating the concentration of IL-1*β* concentration (primary outcome) in 20 saliva samples by enzyme-linked immunosorbent assay (ELISA), the sample size was calculated according to the following equation:

(1) $$\begin{array}{c} \text{Sample size}=r+1/r\;\times \;\left(\text{SD}\right)2\;\times \;\left(Z\beta +\;Z\alpha /2\right)2/d2, \end{array}$$

where *r* was cases/controls ratio ( = 1); SD was the standard deviation (65.7); *Zβ* was the desired power of 90% ( = 1.28); *Zα*/2 was 5% type 1 error ( = 1.96); *d* was the expected mean difference between case and control ( = 53.4). Accordingly, a sample of 37 participants in each group was estimated. To boost the power of the study, a higher sample size was considered (45 for the healthy control group, 51 for the unstable periodontitis group, and 39 for the stable periodontitis group).

### 2.2. Clinical Parameters

After participants selection, all clinical periodontal parameters, including the plaque index (PI) [27], BOP [28], PPD, and CAL, were recorded using a Michigan O periodontal probe (MEDESY, Maniago, Italy) by the same calibrated examiner. Plaque-disclosing pellets (Guided Biofilm Therapy, biofilm discloser, Zwingenberg, Germany) were used to aid in the detection of the dental biofilm. BOP, PPD, and CAL were recorded at six sites/tooth, while the PI was recorded at four sites/tooth.

Prior to starting the study, the examiner was calibrated for diagnosis and recording of clinical parameters with 2-h intervals between two readings. The calibration sessions were repeated on different days until reaching an accepted level of agreement between readings; >0.75 kappa values for PI and BOP as well as >0.9 intraclass correlation coefficient values for PPD and CAL.

### 2.3. Salivary Sample Collection and Biochemical Analysis

Before recording the clinical parameters, unstimulated saliva samples were collected from participants, as previously reported [29]. From 9 AM to 12 PM, participants were asked to refrain from drinking, eating, and brushing their teeth 1 h before sample collection. Then, each participant was asked to rinse his/her mouth with tap water to remove any food residue, and a saliva sample was collected 10 min later. The participants were asked to sit comfortably in an upright position with their heads tilted slightly downwards. The participants were instructed to allow saliva to pool on the floor of their mouths before gently expectorating into a graduated sterile tube for 5 min to obtain 1 ml of unstimulated saliva. Then, the tube was centrifuged for 20 min at room temperature at 1000 x *g* (800 electrical centrifuge—China). A total of 500 μl of the supernatant was transferred into an Eppendorf tube (DNase/Nase free, BIOFL) to estimate the concentrations of IL-1*β* and IL-10. All samples were saved at −80°C in a deep freezer (Angelantoni Life Science, UK).

At the time of sample analysis, frozen saliva samples were allowed to thaw at room temperature. The concentrations of IL-1*β* and IL-10 in the samples were determined using ELISA kits (Cloud-Clone Corp, USA; IL-1*β*: SEA563Hu; IL-10: SEA056Hu) according to manufactural instructions. Standard curves were plotted, and regression equations were derived to convert the optical density values of IL-1*β* and IL-10 in the samples to their respective quantities in picograms per milliliter (pg/ml).

### 2.4. Statistical Analysis

The distribution of continuous variables was checked using the Shapiro–Wilk test. Mean ± SD was used to describe the continuous data, while the frequency and percentage were used for describing categorical variables. Kruskal–Wallis test was used to compare among groups with Bonferroni post hoc analysis for pairwise comparisons. While the Mann–Whitney *U*-test was used for comparing two groups. Categorical variables were analyzed using Chi-square test (*X*^2^). The Correlations between clinical parameters and biochemical variables were performed using Spearman's coefficient correlation test. Binary logistic regression analysis was applied to explore the association of IL-1*β* and IL-10 as independent variables to the study groups, which was adjusted by age. The receiver operating characteristic curve (ROC) and area under the curve (AUC) were used to explore the sensitivity, specificity, and cutoff values for each biomarker, as well as the ratio between the biomarkers for discriminating between groups. The Youden index was used to determine the optimal cutoff value for each tested biomarker [30]. The latter statistical test was accomplished after dichotomizing the concentration of each biomarker as “0” for health and “1” for periodontal disease. All the statistical analyses of the data were performed and processed with GraphPad Prism software (version 9.0). Statistical difference was considered significant when *p*  < 0.05.

## 3. Result

A total of 135 participants were recruited for this study who were distributed into healthy control (*n* = 45), stable periodontitis (*n* = 39) and unstable periodontitis (*n* = 51) groups. The participants in the healthy control group were younger than participants in the other groups. The distribution of sex and mean BMI were not significantly different among the groups. The basic characteristics of the study sample are shown in Table 1. Furthermore, PI and BOP were significantly higher in unstable periodontitis than in other groups. However, no significant difference was detected between the healthy control group and the stable periodontitis group. Deeper pockets were observed in the unstable periodontitis group compared to the stable periodontist group (*p*  < 0.05). On the other hand, no significant difference was found between periodontitis groups regarding CAL measurements (Table 1).

**Figure 1:**
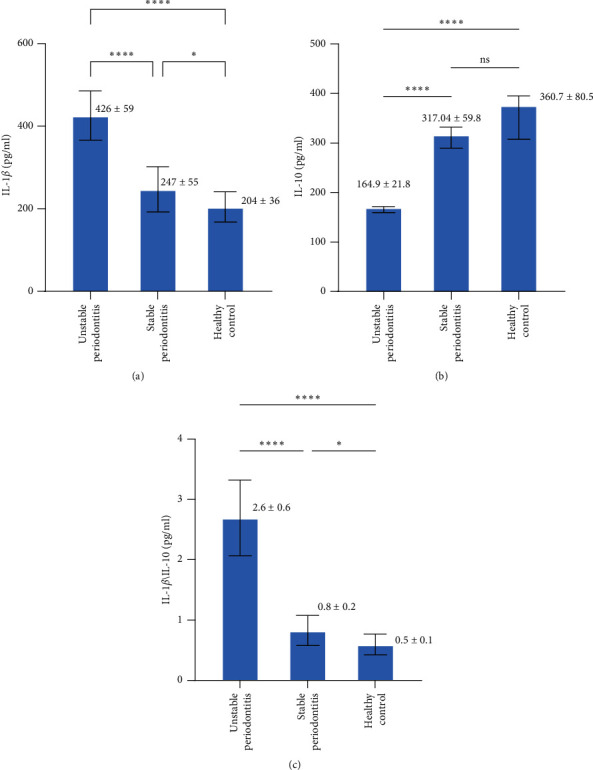
Salivary concentrations of (A) IL-1*β*, (B) IL-10, and (C) IL-1*β*/IL-10 ratio. The levels of IL-1*β*, and IL-1*β*/IL-10 ratio were significantly higher in participants with periodontitis than in healthy periodontium. The level of IL-10 was significantly lower in participants with unstable periodontitis than in healthy periodontium; however, IL-10 did not show any significant difference between salivary samples of participants with stable periodontitis and healthy. *p* value  ^*∗*^<0.05,  ^*∗∗∗∗*^<0.001. IL, interleukin.

A significantly higher salivary level of IL-1*β* was detected in the unstable periodontitis group (426 ± 59 pg/ml) than in the stable periodontitis group (247 ± 55 pg/ml; *p*  < 0.001) and healthy control group (204 ± 36 pg/ml; *p*  < 0.001) (Figure 1A); moreover, the level was significantly higher in the stable periodontitis group than healthy control group (*p*  < 0.05). In contrast, the lowest salivary level of IL-10 was detected in the unstable group (164 ± 21.8 pg/ml) compared to the other groups. No significant difference (*p*  > 0.05) in the salivary level of IL-10 was found between the stable periodontitis group (317.04 ± 59.8 pg/ml) and healthy control group (360.7 ± 80.5 pg/ml), as shown in Figure 1B. The ratio between the above-mentioned biomarkers was found to be significantly different among the groups (*p*  < 0.001); higher in unstable periodontitis group followed by stable periodontitis and healthy control groups (Figure 1C).

After adjusting the effect of age, an increased salivary level of IL-1*β* was found to be associated with an increased probability to have periodontitis (odd ratio = 1.033; *p* = 0.004). Obviously, an increase by one unit of this marker was associated with the likelihood of the incidence of unstable (odd ratio = 1.979) and stable (odd ratio = 1.989) periodontitis from health, respectively. Similarly, an elevated level of IL-1*β* was also detected to be significantly associated (*β* coefficient = 0.079; *p* = 0.004) with the incidence of unstable from stable periodontitis. Such relations were found not significant for IL-10 (Table 2). However, both IL-1*β* and IL-10 were found to be positively and negatively (respectively) associated with the clinical periodontal parameters. While the correlation between the levels of salivary IL-1*β* and IL-10 was negative and significant, as illustrated in Table 3.

**Figure 2:**
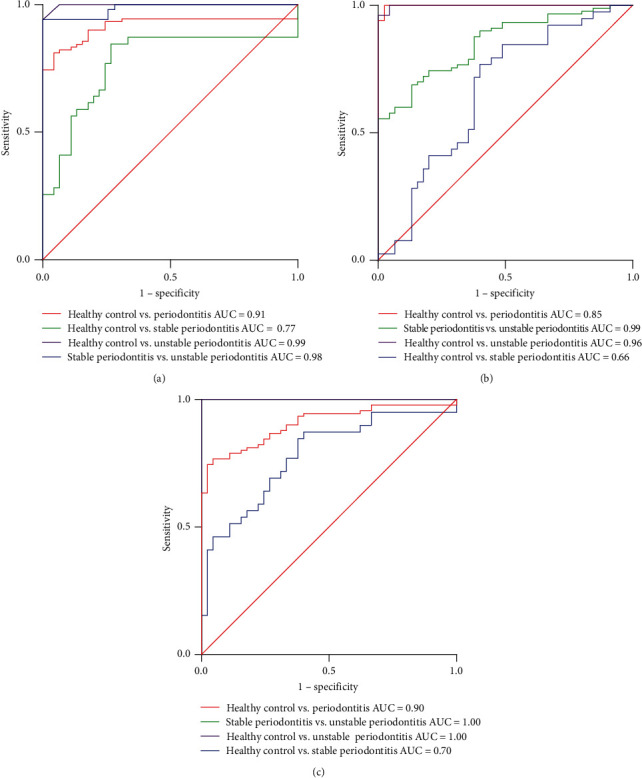
Receiver operating characteristic of salivary biomarkers (A) IL-1*β*, (B) IL-10, and (C) IL-1*β*/IL-10. All biomarkers showed high accuracy in distinguishing periodontal health from periodontitis status. The ratio of IL-1*β*/IL-10 showed the highest potential to differentiate periodontal health from unstable periodontitis (AUC: 1) and stable periodontitis from unstable periodontitis (AUC: 1), while periodontal health from stable periodontitis (AUC) showed the lowest value (AUC: 0.77). Additionally, all salivary biomarkers showed moderately good accuracy in discriminating between periodontal health from stable periodontitis (IL-1*β* AUC: 0.77 and IL-10 AUC: 0.66) AUC, the area under the curve; IL, interleukin.

Table 4 and Figure 2 illustrate the ROC curve, AUC, sensitivity, specificity, and cutoff values of salivary biomarkers and their ratio to differentiate periodontal health from periodontitis and discriminate the current status of periodontitis. Both IL-1*β* and IL-10 biomarkers, as well as their ratio, showed high sensitivity and specificity and excellent potential to discriminate periodontal heath from unstable periodontitis (AUC = 0.99, 0.96, and 1.00) and stable periodontitis from unstable periodontitis (AUC 0.98, 0.99, and 1.00), respectively. Similarly, they exhibited the same pattern in discriminating periodontal heath from periodontitis (stable and unstable) (AUC = 0.91, 0.85, and 0.90, respectively). These salivary biomarkers and their ratio showed an accepted potential to discriminate between periodontal health and stable periodontitis.

Interestingly, the IL-1*β*/IL-10 ratio showed an improved diagnostic potential (AUC of 1, *p*  < 0.0001) to discriminate between periodontal health from unstable periodontitis (cutoff value >1.35) and stable periodontitis from unstable periodontitis (cutoff value >1.55) (Table 4).

## 4. Discussion

In the current study, analysis of the data showed that salivary IL-1*β*, IL-10, and IL-1*β*/IL-10 ratio had high accuracy in differentiating periodontal health from periodontitis. Also, they demonstrated high sensitivity and specificity to discriminate unstable periodontitis from stable periodontitis and periodontal health. This case–control study aims to assess the potential accuracy of the aforementioned salivary proteins and their ratio as diagnostic biomarkers.

In the recent classification of periodontal diseases, a new entity called stable periodontitis was introduced, which defines a periodontium that has restored healthy status after successful periodontal therapy. It could be differentiated from unstable periodontitis either by the presence of PPD >4 mm or PPD equal to 4 mm, which shows bleeding during examination [20]. Using manual probing to measure periodontal parameters is not a sensitive procedure. For instance, forceful probing could induce bleeding or incorrectly record a deeper pocket depth. Such issues are not uncommon during probing [31]. A lack of accuracy in identifying stable from unstable periodontium leads to drastically changing treatment plans from supportive periodontal therapy to active periodontal therapy. Therefore, a precise diagnosis is essential for customizing the treatment approach appropriately [32]. In the past several decades, using biomarkers found in oral fluid to distinguish between periodontal health and disease has shown promising findings [21].

In this study, saliva has been chosen as a biomarker source due to the ease of collection and handling as well as obtaining adequate volumes of samples. Additionally, salivary biomarkers reflect all local and systemic conditions and are suited for screening purposes [33–35]. Recently, point-of-care (POC) mouth rinse tests, such as the active MMP-8 POC test, have been incorporated into the new classification system for periodontitis [36]. Although they are rapid, sensitive, specific, and robust, they are costly to use in routine clinical practice compared to traditional diagnostic methods [32]. This study exclusively used biomarkers with clear evidence of correlation with the pathogenesis of periodontal disease.

In this study, IL-1*β* was chosen as a robust diagnostic biomarker to discriminate healthy from diseased periodontal tissue. It is strongly linked to the severity and progression of periodontal diseases, tooth loss, and tissue response to periodontal therapy [37, 38]. IL-1*β* has been observed to upregulate the expression of many cytokines involved in the degradation of tissues and bone resorption, such as collagenases and prostaglandins [37, 38]. Furthermore, IL-1*β* increases the expression of adhesion molecules, which in turn exacerbates inflammatory reactions and increases the extent of tissue degradation by promoting the recruitment of inflammatory cells [39]. It is not surprising that salivary levels of IL-1*β* were increased in the periodontitis groups in this study, as previously reported [40–43]. Accordingly, salivary IL-1*β* has been reported to exhibit a potential to distinguish periodontitis from periodontal health with varying sensitivity (54%–88%) and specificity (52%–100%) [41, 44–46]. These previous reports agree with the current study findings. Moreover, this explanation might also interpret the significantly higher level of IL-1*β* in unstable periodontitis than in stable periodontitis and its accuracy in discriminating these two periodontitis statuses.

IL-10 is a potent anti-inflammatory mediator that suppresses both immunoproliferative and inflammatory reactions. IL−10 inhibits the production of cytokines, such as IL-1, IL-6, and TNF-*α*, by T-helper1 cells and suppresses the production of collagenase, gelatinase, and nitric oxide [18, 47]. In this study, the level of IL-10 was lower in periodontitis patients than in peers with healthy periodontium; this finding is in accordance with what has been reported in the literature [48–50]. Scarel-Caminaga et al. proposed that individuals who produce a lot of IL-10 might be more resistant to periodontitis due to its anti-inflammatory effect [51]. Moreover, IL-10 levels have been reported to be elevated 2 weeks [52], 6 weeks [53], and 2 months [54] after nonsurgical periodontal therapy. Therefore, a higher salivary level of IL-10 was expected in subjects with healthy periodontium or periodontitis patients who had completed periodontal therapy, i.e., stable periodontitis. However, contrary findings have also been reported: periodontitis patients have higher levels of IL-10 than healthy controls [18, 55–57]. This controversy encourages the authors to investigate the salivary level of this biomarker in this study.

In this study, the IL-1*β*/IL-10 ratio exhibited high sensitivity and specificity for potentially discriminating periodontitis from healthy groups as well as for distinguishing unstable from stable periodontitis groups. No doubt, tissue affected by periodontitis is associated with an imbalance between pro and anti-inflammatory cytokines (IL-17, IL-23, IL-6, IL-8, TNF-*α*, and interferon-*γ*) in favor of pro-inflammatory burden [48, 49, 58]. These findings indicate that the combination of biomarkers not only enhances the accuracy of diagnosis compared with a single biomarker but also attains sufficient reliability for diagnosing periodontitis.

The concentration of salivary IL-1*β* was found to be positively associated with clinical periodontal parameters (PL, BOP, PPD, and CAL), as previously reported [18, 41, 59, 60]. This significant correlation highlights the essential function of IL-1*β* in the host immune response related to periodontitis, particularly in patients with identified IL-1*β* gene variants [61, 62]. Interestingly, the means of BOP and PPD were significantly different between stable and unstable periodontitis patients. One can conclude that IL-1*β* is more related to the BOP and PPD parameters, which could discriminate among periodontitis statuses. Furthermore, salivary IL-10 was found to be negatively related to BOP, PPD, and CAL. This finding is not surprising as IL-10 is a potent anti-inflammatory cytokine [18, 47] and is expected to be elevated when clinical periodontal parameters are improved, as previously reported [10, 18, 63].

As a limitation, smokers and patients having systemic diseases were excluded in this study. This fact impacts the external validity of this study, as its findings cannot be generalized to the population. However, excluding such individuals was necessary to eliminate biases and confounding factors. Larger-scale studies involving participants without exclusion are highly suggested. Another limitation is that this study only assessed host-derived salivary biomarkers while integrating bacterial and host-derived salivary biomarkers might provide a better realizable diagnostic approach.

## 5. Conclusion

Despite the limitations of this study, salivary IL-1*β* and IL-10 potentially possess a high diagnostic power for discriminating periodontal health from periodontitis as well as for distinguishing stable from unstable periodontitis. This diagnostic power is further increased when IL-1*β*/IL-10 is employed.

## 6. Clinical Relevance

### 6.1. Scientific Rationale for the Study

The conventional periodontitis diagnostic approach is based on clinical parameters that are inaccurate in detecting early periodontitis, and they could potentially mirror the previous tissue damage. Salivary biomarkers have been found to have the potential to enhance screening, diagnosis, and monitoring of periodontitis.

### 6.2. Principle Findings

IL-1*β* and IL-10 have great diagnostic capabilities when used separately or in combination for the diagnosis of periodontitis.

### 6.3. Practical Implications

The IL-1*β*/IL-10 ratio in saliva exhibits a valuable potential for diagnosing periodontitis and in discriminating periodontitis stability.

## Figures and Tables

**Table 1:** Basic characteristics of the study sample.

Variables	Healthy control (45)	Stable periodontitis (39)	Unstable periodontitis (51)	*p* Value
Age (mean ± SD)^a^	34.33 ± 4.32	53.73 ± 7.87	55.25 ± 8.45	**<0.001**
Sex (*n*, %)^b^				
Male	26, 57.8%	24, 61.5%	35, 68.6%	0.534
Female	19, 42.2%	15, 38.5%	16, 31.4%	
BMI (mean ± SD)^a^	23.42 ± 1.19	23.88 ± 1.11	23.87 ± 1.20	0.07
PI (mean ± SD)^a^	0.37 ± 0.19	0.40 ± 0.26	0.94 ± 0.12	**<0.001**
BOP (mean ± SD)^a^	0.04 ± 0.02	0.05 ± 0.02	0.79 ± 0.21	**<0.001**
PPD_mm_ (mean ± SD)^a^	<3	3.46 ± 0.44	4.71 ± 0.62	**<0.001**
CAL_mm_ (mean ± SD)^a^	0	3.08 ± 1.02	3.38 ± 0.91	0.06

Abbreviations: BOP, bleeding on probing; CAL, clinical attachment loss; *n* (%), number (percent); PI, plaque index; PPD, probing pocket depth, only sites with PPD >3 mm were recorded; SD, standard deviation.

^a^Comparison by Kruskal–Wallis test and pairwise post hoc; significance was considered when *p*  < 0.05 (bold). The mean age of the healthy group was significantly less than other groups; no significant differences between the ages of stable and unstable patients were found. The mean body mass index (BMI) was not significantly different among the study groups. There were no significant differences between the means of PI and BOP of healthy control and stable periodontitis patients. Both healthy control and stable periodontitis groups had significantly lower means of PI and BOP than unstable periodontitis group.

^b^Comparison by *X*^2^ test, significance was considered when *p*  < 0.05 (bold).

**Table 2:** Binary regression for independent variables as predictors for periodontitis stability adjusted by age.

Groups (reference)	Predictor variables	Effects (*β*)	Odd ratio	95% Cl for odd ratio	*p* Value ^*∗*^
Periodontitis (healthy group)	IL-1*β*	0.032	1.033	1.010–1.056	**0.004**
	IL-10	−0.450	0.638	0.355–1.14	0.99

Unstable periodontitis (healthy group)	IL-1*β*	0.021	1.979	1.972–1.986	**<0.001**
	IL-10	−0.014	0.987	0.981–0.992	**<0.001**

Stable periodontitis (healthy group)	IL-1*β*	0.160	1.989	0.959–2.01	**0.022**
	IL-10	−0.014	0.986	0.968–1.004	0.119

Unstable periodontitis (stable periodontitis)	IL-1*β*	0.079	1.924	1.875–1.975	**0.004**
	IL-10	−0.162	0.851	0.683–1.023	0.05

*Note*: IL-1*β* was associated with an increased probability to have unstable and stable periodontitis from health as well as unstable from stable periodontitis. While IL-10 was only associated with decreasing the probability of developing unstable periodontitis from health.

Abbreviations: *β*, logistic regression coefficient; CI, confidence interval; IL, interleukin.

^*∗*^Significance at *p*  < 0.05 (bold).

**Table 3:** Correlations between salivary biomarkers and clinical periodontal parameters.

Salivary biomarker		PI	BOP	PPD	CAL	IL-1*β*	IL-10
IL-1*β*	*r*	0.629	0.852	0.798	0.696	NA	−0.711
	*p* Value ^*∗*^	**<0.001** ^*∗∗*^	**<0.001** ^*∗∗*^	**<0.001** ^*∗∗*^	**<0.001** ^*∗∗*^	NA	**<0.001** ^*∗∗*^

IL-10	*r*	−0.651	−0.750	−0.764	−0.571	−0.711	NA
	*p* Value ^*∗*^	**<0.001** ^*∗∗*^	**<0.001** ^*∗∗*^	**<0.001** ^*∗∗*^	**<0.001** ^*∗∗*^	**<0.001** ^*∗∗*^	NA

*Note:* The clinical periodontal parameters were associated with IL-1*β* (positively) and IL-10 (negatively). The correlation between the levels of salivary IL-1*β* and IL-10 was negative and significant.

Abbreviations: BOP, bleeding on probing; CAL, clinical attachment loss; IL, interleukin; NA, not applicable; PI, plaque index; PPD, probing pocket depth, only sites with PPD >3 mm were recorded; *r*, correlation coefficient.

^*∗*^Significance at *p*  < 0.05 and  ^*∗∗*^*p*  < 0.001 (bold) by Spearman's correlation assay.

**Table 4:** Area under the curve (AUC), sensitivity, specificity, and cutoff values for study groups.

Salivary biomarker	AUC	Cutoff value (pg/ml)	Sensitivity	Specificity	95% CI	*p* Value ^*∗*^
Healthy vs. periodontitis						
IL-1*β*	0.91	>256.0	0.81	0.80	0.84–0.95	**<0.0001**
IL-10	0.85	<288.6	0.70	0.80	0.78–0.91	**<0.0001**
IL-1*β*/IL-10 ratio	0.90	>0.73	0.81	0.80	0.71–0.88	**<0.0001**
Healthy vs. unstable periodontitis						
IL-1*β*	0.99	>285.4	0.98	0.95	0.99–1.00	**<0.0001**
IL-10	0.96	<216.2	1.00	0.95	0.99–1.00	**<0.0001**
IL-1*β*/IL-10 ratio	1.00	>1.35	1.00	1.00	1.00–1.00	**<0.0001**
Healthy vs. stable periodontitis						
IL-1*β*	0.77	>217.1	0.84	0.73	0.66–0.88	**<0.0001**
IL-10	0.66	<352.0	0.76	0.61	0.54–0.77	**<0.01**
IL-1*β*/IL-10 ratio	0.70	>0.6106	0.87	0.60	0.73–0.94	**<0.0001**
Stable periodontitis vs. unstable periodontitis						
IL-1*β*	0.98	>307.1	0.94	0.94	0.96–1.00	**<0.0001**
IL-10	0.99	<223.8	1.00	0.97	0.99–1.00	**<0.0001**
IL-1*β*/IL-10 ratio	1.00	>1.55	1.00	1.00	1.00–1.00	**<0.0001**

Abbreviations: CI, confidence interval; IL, interleukin.

^*∗*^Significance at *p*  < 0.05 (bold).

## Data Availability

The data that support the findings of this study are available from the corresponding author upon reasonable request.
